# Evaluation of Antimalarial Activity of the 80% Methanolic Stem Bark Extract of *Combretum molle* Against *Plasmodium berghei* in Mice

**DOI:** 10.1177/2515690X19890866

**Published:** 2019-12-03

**Authors:** Tafere Mulaw, Muluken Wubetu, Bekalu Dessie, Gebreselassie Demeke, Yalew Molla

**Affiliations:** 1 University of Gondar, Gondar, Ethiopia; 2 Debre Markos University, Debre Markos, Ethiopia

**Keywords:** *Combretum molle*, antimalarial, folklore use, chemosuppressive, crude extract

## Abstract

**Background.:**

People are dependent on the traditional use of medicinal plants for the treatment of malaria without scientific validations. Therefore, this study aimed to evaluate the antimalarial activity of methanolic stem bark extract of *Combretum molle* in mice.

**Methods.:**

After being infected with *Plasmodium berghei*, the mice were randomly divided into 5 groups (n = 5). In all cases, group I mice were treated as negative control and received 3% Tween 80; group II mice were treated with 25 mg/kg chloroquine; and groups III, IV, and V mice were treated with 100 mg/kg, 200 mg/kg, and 400 mg/kg of crude extract, respectively. Data were analyzed using one-way analysis of variance followed by Tukey’s post hoc analysis.

**Results.:**

In the chemosuppressive test, the parasitic suppression effect of the crude extract was found to be significant (*P* < 0.05) as compared with the negative control. In the curative experiment, the average parasitic level of those mice treated by the 3 doses of the crude extract was significantly suppressed at days 5, 6, and 7 of treatment (*P* < 0.001). Besides, the crude extract had been found to have a chemoprophylactic role as it inhibited the parasite level significantly relative to the negative control (*P* < 0.001). Moreover, the crude extract had preventive effects on packed cell volume reduction in the 3 tests (*P* < 0.001).

**Conclusions.:**

The findings of the present study has supported the folklore use of the leaves of *Combretum molle* in the treatment of malaria. Therefore, further fractionation and characterization of the crude extract is necessary to identify the responsible lead compound(s) responsible for antiplasmodial activity.

## Introduction

Malaria is a febrile hemolytic disease caused by plasmodium species including *Plasmodium falciparum*, *Plasmodium vivax*, *Plasmodium malariae*, *Plasmodium ovale*, and *Plasmodium knowlesi*, which are recognized to infect and cause clinical malaria in humans.^1^

According to the World Malaria Report 2018, there were 219 million cases of malaria globally in 2017 and 435 000 malaria deaths. The burden was heaviest in the African Region, where an estimated 93% of all malaria deaths occurred, and in children aged under 5 years, who accounted for 61% of all deaths.^2^ Malaria is ranked as the leading communicable disease in Ethiopia. About 75% of the geographic area of the country has significant malaria transmission risk (defined as areas <2000 m in altitude).^3^

Different countries of the world, including Africa, are dependent on traditional medicine to meet their primary health care needs. For instance, up to 80% of the Ethiopian population uses traditional practice as a primary source of health care.^4^ In spite of the high prevalence of using traditional remedies, there is poor integration of traditional medicine use and modern medicine in the clinical and medical practices, particularly in sub-Saharan countries.^5^

The treatment of malaria is achieved by using standard drugs or using traditional preparations of medicinal plants. The management of malaria using standard drugs is subjected to antimalarial drug resistance. Resistance to antimalarial medicines has been documented in all classes of antimalarials, including the artemisinin derivatives where discovering new compound(s) from medicinal plants with a potential antimalarial activity is necessary.^6–8^

Traditional preparations of medicinal plants have in the past been the source of some of the most successful antimalarial agents such as the quinine and artemisinin derivatives,^9–11^ which encouraged researchers to conduct researches on medicinal plants for identifying a responsible lead compound with the potential of treating malarial diseases. However, the folklore use of medicinal plants lacks scientific evidence on safety, dosage to be used, and efficacy of the practices.^11^

It is estimated that about 80% of the Ethiopian population rely on medicinal plants for treating various illnesses including malaria.^11^ *Combretum molle* is one of the medicinal plants that has been used traditionally for the treatment of different diseases in east and west Africa including malarial, protozoal, and bacterial infections.^12^ In Ethiopia, the plant is traditionally claimed to be used for the treatment of different diseases including malaria. The soaked forms of leaf and the stem bark powder are used for the treatment of malaria.^13^ The plant is also traditionally claimed to be used for the treatment of tongue infections and diarrheal disease.^14,15^

The different parts of the studied plant have been scientifically approved to have different pharmacological activities such as anti-inflammatory,^16^ antischistosomal, anthelmintic,^17^ antimicrobial,^18,19^ and antifungal activities.^20,21^ The antiplasmodial activity of the seed (in vivo) and stem bark (in vitro) extract had also been approved pharmacologically.^22,23^

Nevertheless, the traditional antimalarial efficacy and safety of the stem bark of *Combretum molle* in vivo are not yet validated scientifically. Therefore, the purpose of the present study was to evaluate the in vivo antimalarial activity of the crude stem bark extract of *Combretum molle* in mice.

## Materials and Methods

### Plant Material

The stem bark of *Combretum molle* was collected in October 2016 from the area around Debre Markos town, which is located in Amhara Regional Government 300 km northwest of Addis Ababa, Central Ethiopia. The authentication of the plant material was confirmed by the National Herbarium, Department of Biology, Addis Ababa University, where voucher specimen TM001 was deposited.

### Experimental Animals

Swiss albino mice of either sex weighing 24 to 30 g and age 6 to 8 weeks were purchased from Ethiopian Public Health Institute animal house, Addis Ababa. All animals were housed in an air-conditioned room and allowed to acclimatize for 1 week before the study. The animals were kept at room temperature and exposed to a 12 hour light/dark cycle. All the experiments were conducted following the internationally accepted laboratory animal use and care guidelines.^24^ Before and during the experiment, the mice were allowed free access to standard pellets and water ad libitum.

### Parasite

*Plasmodium berghei* ANKA strain (chloroquine sensitive) was brought from Aklilu Lemma Institute of Pathobiology. Then, the parasite was maintained in the laboratory by serial blood transfusion from one mouse to another mouse on a weekly basis.

### Extract Preparation

Fresh stem bark of the plant material was gently washed with distilled water to remove dirt and soil, and dried under shade at optimal ventilation for 2 weeks. The dried stem bark was further chopped into small pieces and reduced to coarsely sized powder. The coarsely powdered bark was subjected to maceration procedure using 80% methanol. The plant and solvent mixture was placed on an orbital shaker (at 160 rpm) for 72 hours at room temperature. Then, each sample was filtered out using a Whatman Filter Paper No. 1. Then, the filtrate was concentrated in a rotary evaporator at a temperature of 40°C. Finally, the weight of the dry extract was measured after which it was packed separately in a screw-capped glass bottle and stored in a deep freezer.

### Preliminary Phytochemical Screening

The 80% methanol extract of *Combretum molle* was screened for the presence of alkaloids, flavonoids, polyphenols, tannins, saponins, triterpenoids, and steroidal compounds using standard procedures.^25–28^

### Acute Oral Toxicity Test

Female Swiss albino mice were used for acute oral toxicity study. Oral toxicity study was conducted as per the internationally accepted protocol drawn under OECD guidelines 425: 2008.^29^ Nine mice were randomly divided into 3 groups of 3 mice per cage. The animals were physically active and regularly consumed food and water.

Before the administration of a single dose of the extract, the mice were fasted for 2 hours. Then, the mice in the first group and the second group were given distilled water and the 80% stem bark extract of *Combretum molle* 2 g/kg dissolved in distilled water orally, respectively, and the mice in the third group were provided the dissolved 80% methanol extract at 5 g/kg after following the first 2 groups for 14 days.

The mice were observed continuously for 1 hour after administration of the extract; intermittently for 4 hours, over 24 hours and for 14 days. Gross behavioral changes such as loss of appetite, hair erection, lacrimation, tremors, convulsions, salivation, diarrhea, mortality, and other signs of toxicity manifestation were observed.

### Four-Day Suppressive Test (Activity on Early Infection)

The chemosuppressive test was done using the standard 4-day suppressive test against *Plasmodium berghei* infection in mice. The mice were randomly divided into 5 groups with 5 mice each. The 3 treatment groups (III, IV, and V) were provided with 100 mg/kg, 200 mg/kg, and 400 mg/kg doses of the plant extract. The positive control groups received 10 mg/kg of chloroquine (positive control group) and 10 mL/kg of distilled water (negative control group). Treatment was started 3 hours after infecting the mice with infected blood containing about 1 × 10^7^ *Plasmodium berghei* and was then continued for 4 consecutive days.^30–32^

On the fifth day, thin blood smears from the tail vein of each mouse was prepared on microscopic slides. The blood film was fixed with absolute methanol and stained with 10% Giemsa solution at pH 7.2 for 15 minutes. The slides were taken out and dried with the room temperature. The number of parasitized RBCs (red blood cells) was counted under a microscope. The percentage suppression of parasitemia was calculated for each test concentration by comparing the parasitemia in infected controls with those groups that received different concentrations of the test extract. Compounds reducing parasitemia by ≥30% were considered as active. Percent parasitemia and percent parasitemia suppression was calculated by using the following formula^30^:

$$\text{\% Parasitemia }=\frac{\text{Number of parasitized RBC }}{\text{Total number of RBC count}}\times 100$$

$$\text{\% Suppression}=\frac{(\text{Mean parasitemia of negative control}-\text{Mean parasitemia of treated groups})\;}{\text{ Mean parasitemia of negative control}}\times 100$$

### Curative/Rane Test (Activity on Established Infection)

Evaluation of the curative antimalarial potential of the extract was done by using a method described previously.^31,32^ On the first day, blood containing approximately the inoculum of 1 × 10^7^ *Plasmodium berghei* was injected into 25 Swiss Albino mice, intraperitoneally (IP). After 72 hours, the mice were randomly divided into 5 groups of 5 mice each. Subsequently, 3 different doses of the extract (100 mg/kg, 200 mg/kg, and 400 mg/kg/day), chloroquine phosphate (25 mg/kg/day), and 3% Tween-80 in distilled water (1 mL/100 g/day) were given orally to the respective groups. These treatments were administered once daily for 3 consecutive days. For each mouse, thin blood film stained with 10% Giemsa was prepared from tail blood of each mouse daily for 5 consecutive days to monitor the levels of parasitemia. The extract is considered active when parasitemia was reduced by ≥30%. The mice were further observed for 30 days. Any death that occurred during this period was recorded and used to determine the mean survival time. Similar to the chemosuppressive test, the average % parasitemia suppression was calculated.

### Repository/Prophylactic Activity (Activity on Residual Infection)

Evaluation of prophylactic potential of the active compounds in a 4-day suppressive test of the extract was done by methods described previously.^31,32^ Twenty-five Swiss Albino mice were randomly divided into 5 groups of 5 mice each. They were administered orally with 100, 200, and 400 mg/kg/day of the extracts, chloroquine phosphate (25 mg/kg/day), and 3% Tween-80 in distilled water (1 mL/100 g/day) for 3 consecutive days of the respective groups. On the fourth day, a standard inoculum of 1 × 10^7^ *Plasmodium berghei* infected-erythrocytes was administered by the IP route to each mouse. After 72 hours (on the seventh day), thin blood smears were prepared from the tail blood. Percentage parasitemia and the percentage of chemosuppression of parasitemia were calculated using the formula described in the chemosuppressive test.

### Determination of Mean Survival Time

Mean survival time (MST) is a parameter that is commonly used to evaluate the efficacy of antimalarial plant extracts. For each group, in each test, MST was determined by the average survival time (days) of the mice (post-inoculation) in 30 days post-infection. A dose that results in survival time greater than that of infected nontreated mice was considered as active. Death occurring before day 5 of infected and treated mice was regarded as toxic death. The parasitemia level of the animals that survived after the 30 days was determined from thin blood film.^31,32^ The MST of each group was calculated as

$$\text{MST }=\;\frac{\text{Sum of survival time of all mice in a group }(\text{days})}{\text{Total number of mice in that group}}$$

### Body Weight Determination

Similarly, body weight loss is one feature of rodent malaria infections.^31^ Body weight of each mouse was measured to determine the effectiveness of the extract. The body weight of each mouse in all groups was taken before infection (day 0) and on day 4. Each mouse in a group was measured using a sensitive balance. Then, the average percent change in body weight was compared with the control groups.

### Determination of Packed Cell Volume

Packed cell volume (PCV) was measured to predict the effectiveness of the test extracts in preventing hemolysis resulting from increasing parasitemia associated with malaria.^31^ Blood was collected from the tail of each mouse in heparinized micro hematocrit capillary tubes by filling three fourths of its volume. The tubes were sealed by sealant and placed in a micro hematocrit centrifuge with the sealed ends outwards. Then, the blood was centrifuged at 12 000 rpm for 15 minutes. The PCV of each mouse was then measured before infection and on day 4 after infection using the following formula:

$$\text{PCV }=\;\frac{\text{Volume of erythrocytes in a given volume of blood}}{\text{Total blood volume}}$$

### Data Analysis

Data were analyzed using SPSS version 20 software. The one-way analysis of variance followed by Tukey’s HSD post hoc test was used to compare means among and within groups. The statistical analysis was considered significant when *P* < 0.05.

### Data Quality Control

Data quality was controlled by the use of the appropriate number of animals, randomization, coding, and using standard instruments and chemicals of analytical grade.

## Results

### Phytochemical Analysis

Preliminary phytochemical study of the stem bark extract of *Combretum molle* revealed the presence of alkaloids, anthraquinone, cardiac glycosides, terpenoids, flavonoids, phenols, and saponins but not tannins (Table 1).

**Table 1.** Phytochemical Constituents of Methanol Extract of the Stem Bark of *Combretum molle*.

Secondary Metabolite	Test	Test Result
Alkaloids	Mayer’s test	Positive
Antraquinone	Ninhydrin test	Positive
Terpinoids	Copper acetate test	Positive
Flavonoids	Shinoda’s test	Positive
Phenols	Ferric chloride test	Positive
Tannins	Lead acetate test	Negative

### Acute Toxicity Test

The acute toxicity study indicated that the extract did not cause mortality of mice within 24 hours up to 2 g/kg body weight. Gross physical observation of mice revealed no visible signs of acute toxicity like lacrimation, hair erection, tremors, convulsions, salivation, and reduction in their motor and feeding activities. Based on these results, the median lethal dose of the extract was found to be greater than 2 g/kg body weight.

### Four-Day Suppressive Test (Activity on Early Infection)

The 4-day suppressive test showed that the crude extract has a significant (*P* < 0.001) inhibitory effect on the average plasmodial parasitemia at all doses (100-400 mg/kg) when compared with negative control. The crude extract at a dose of 400 mg/kg revealed the highest percentage parasite suppression activity (59.7%), followed by 200 mg/kg (52.5%) and 100 mg/kg (38.6%) (Table 2). But the standard drug, chloroquine, has a chemosuppression of 100%.

**Table 2.** Chemosuppressive Activity of Methanolic Crude Extract of Stem Bark of *Combretum molle* against *Plasmodium berghei* Infection in Mice.^*^,^†^

Test Dose	Average % Parasitemia	Average % Suppression	Mean Survival Time ± SEM (Days)
NC	38.95 ± 3.21	0.0	6.2 ± 0.4
CQ 10 mg/kg	0	100	30
CM 100 mg/kg	23.9 ± 1.94^a3^	38.6	8.6 ± 0.5
CM 200 mg/kg	18.5 ± 1.11^a3^	52.5	9.6 ± 0.5^a3b1^
CM 400 mg/kg	15.7 ± 0.93^a3b1^	59.7	13.4 ± 0.8^a3b3^

Abbreviations: CM, *Combretum molle*; CQ, chloroquine; NC, negative control; SEM, standard error of mean.

* Values are expressed as percentage and mean ± SEM (n = 5).

^†^ a, as compared to NC; b, to CM 100 mg/kg; c, to CM 200 mg/kg; d as compared to CM 400 mg/kg; ^1^ *P* < .05; ^2^ *P* < .01; ^3^ *P* < .001.

The middle and the higher doses of the extract had shown to have a more significant survival effect as compared with the negative control and the lower doses of the extract (*P* < 0.05). However, the lower doses of the extract did not reveal a significant survival effect as compared with the negative control (*P* > 0.05; Table 2).

### Body Weight and PCV Variation in Chemosuppressive Test

All groups of mice except those receiving 400 mg/kg of the extract and chloroquine had lost their weight during the chemosuppressive period. The percent change in body weight of mice taking 400 mg/kg of the crude extract and standard drug was statistically different as compared with the negative control, which received the vehicle (*P* < 0.05; Table 3).

**Table 3.** Effect of Crude Methanolic Extract of the Stem Bark of *Combretum molle* on Body Weight and PCV of *Plasmodium Berghei* Infected Mice in Suppressive Test.^*^,^†^

Test Dose	BW D0 ± SEM	BW D4 ± SEM	% BW Change	PCV5 D0 ± SEM	PCV8 D4 ± SEM	% PCV Change
Distilled H_2_O 0.2 mL/NC	27.1 ± 0.84	26 ± 0.8	−3.8	52.2 ± 1.15	44.0 ± 2.5	−15.8
CQ 10 mg/kg	25.9 ± 1.5	26 ± 1.4	0.8^a1b1^	53.6 ± 2.6	52.6 ± 2.4	−1.8^a3^
CM 100 mg/kg	27.7 ± 0.53	26.7 ± 0.67	−3.50	54.4 0.51	50.2 ± 0.66	−7.7
CM 200 mg/kg	26.1 ± 0.84	25.9 ± 0.88	−1.5	51.8 ± 0.58	49 ± 0.63	−5.4^a1^
CM 400 mg/kg	25.3 ± 1.3	25.4 ± 1.5	0.30^a1^	54.8 ± 1.7	53.4 ± 2.0	−2.6^a3^

Abbreviations: BW, body weight; CQ, chloroquine; NC, negative control; PCV, packed cell volume; SEM, standard error of mean.

* Values are expressed as percentage and mean ± SEM (n = 5).

^†^ a, as compared to NC; b, to CM 100 mg/kg; c, to CM 200 mg/kg; d, as compared to CM 400 mg/kg; ^1^ *P* < .05; ^2^ *P* < .01; ^3^ *P* < .001.

The crude extract had also shown a dose-dependent effect on PCV. The decrease in PCV was highest at 100 mg/kg, followed by 200 mg/kg and 400 mg/kg at the end of treatment. The percent reduction in the PCV of mice receiving 400 mg/kg and 200 mg/kg of crude extract, and the standard drug was significantly different as compared with the negative control (*P* < .01; Table 3).

### Curative/Rane Test

At day 4 of treatment, even though their effect was not comparable to the positive control, the middle and the higher doses of the extract revealed a significant curative effect compared with the negative control (*P* < 0.05). At days 5, 6, and 7, all doses of the extract had shown a significant inhibition effect on the plasmodium parasite as compared with the negative control (*P* < 0.01). In addition, on day 6, there were significant parasitemia differences between the lower and the higher doses of the extract (*P* < 0.001) at which the level of parasitemia was significantly lower in those groups taking 400 mg/kg of the crude extract. On day 7, the antiprotozoal effect of 100 mg/kg of the extract was significantly lower than that of the middle and higher doses of the extract (*P* < 0.01; Table 4).

**Table 4.** Effects of *Combretum molle* Stem Bark Extract on Parasitemia in *Plasmodium berghei* Infected Mice in Curative Test.^*^,^†^

Test Dose	Average % Parasitemia (% Suppression)					Mean Survival Time ± SEM (Days)
	Day 3	Day 4	Day 5	Day 6	Day 7	
Distilled water	22.8 ± 3.86	26.4 ± 1.2	35 ± 1.4	40 ± 1.1	45 ± 0.67	7 ± 0.32
CQ 10 mg/kg	18.4 ± 0.93	11.6 ± 2.1^a3^	5.8 ± 1.1^a3^	2.2 ± 0.49^a3^	0.00^a3^	30 ± 0.00^a3^
CM 100 mg/kg	20.3 ± 0.8	21.8 ± 0.7	23 ± 0.71^a3e3^	24.6 ± 0.93^a3e3^	28.2 ± 1.3^a3e3^	9 ± 0.55
CM 200 mg/kg	21.8 ± 1.1	20.8 ± 1.2^a1^	21.6 ± 1.4^a3e3^	21.6 ± 1.2^a3e3^	22.2 ± 1.3^a3e3b2^	9.6 ± 0.51^a1^
CM 400 mg/kg	21.2 ± 1.02	20.8 ± 0.5^a1^	19 ± 0.55^a3e3^	18 ± 0.45^a3e3b3^	18.4 ± 0.8^a3e3b3^	12.2 ± 0.89^a3b2c1^

Abbreviations: CM, *Combretum molle*; CQ, chloroquine; NC, negative control; SEM, standard error of mean.

* Values are expressed as percentage and mean ± SEM (n = 5).

^†^ a, as compared to NC; b, to CM 100 mg/kg; c, to CM 200 mg/kg; d as compared to CM 400 mg/kg; ^1^ *P* < .05; ^2^ *P* < .01; ^3^ *P* < .001.

The survival analysis indicated that the 3 doses of the extract caused a significant increase in the survival of the mice as compared with the negative control (*P* < 0.05). The higher doses of the extract had shown to have a statistically significant survival effect as compared with the middle and the lower doses of the extract (*P* < 0.01; Table 4).

### Body Weight and PCV Variation in the Curative Test

In the curative test model, there were no significant average weight differences among the groups of mice receiving all doses of the crude extract and that of the negative control (*P* > 0.05). Even though the difference in the percent weight change within the groups was not statistically significant, the reduction in the weight of mice receiving the extract and that of the standard drug was minimal as compared with that of the mice in the negative control. Moreover, all doses of the extract and the standard drug had caused a significant change in the percent PCV values as compared with that of the negative control (*P* < 0.05; Table 5).

**Table 5.** Effect of Crude Methanolic Extract of the Stem Bark of *Combretum molle* on Body Weight and PCV of *Plasmodium berghei* Infected Mice in Curative Test.^*^,^†^

Test Dose	BW D0 ± SEM	BW D4 ± SEM	% Change	PCV5 D0 ± SEM	PCV8 D4 ± SEM	% Change
Distilled H_2_O 0.2 mL	22.2 ± 0.96	20.96 ± 1.5	−6.0	52.2 ± 1.31	45.4 ± 0.97	−12.8
CQ 10 mg/kg	21.9 ± 0.94	21.2 ± 0.79	−0.6	54.2 ± 1.53	53.6 ± 0.97	−1.0^a2^
CM 100 mg/kg	26.1 ± 0.74	24.8 ± 0.79	−4.9	55 ± 1.52	52.8 ± 2.2	−4.1^a1^
CM 200 mg/kg	22.6 ± 1.4	21.8 ± 1.4	−3.8	54.6 ± 1.6	52.4 ± 2.3	−4.1^a1^
CM 400 mg/kg	22.5 ± 1	22 ± 0.81	−2.0	54.8 ± 2.2	54 ± 18	−1.3^a2^

Abbreviations: BW, body weight; CM, *Combretum molle*; CQ, chloroquine; NC, negative control; PCV, packed cell volume; SEM, standard error of mean.

* Values are expressed as percentage and mean ± SEM (n = 5).

^†^ a, as compared to NC; b, to CM 100 mg/kg; c, to CM 200 mg/kg; d as compared to CM 400 mg/kg; ^1^ *P* < .05; ^2^ *P* < .01; ^3^ *P* < .001.

### Repository/Prophylactic Activity (Activity on Residual Infection)

In the prophylactic model, all doses of the extract reduced the average percentage of parasitemia significantly (*P* < 0.001) as compared with the negative control. The 400 mg/kg crude extract had also reduced the parasite level significantly (*P* < 0.05) as compared with the 100 mg/kg crude extract. The crude extract at a dose of 400 mg/kg showed the highest percentage parasite suppression activity (54.8%), followed by 200 mg/kg (46.2%) and 100 mg/kg (32.99%). But the standard drug, chloroquine, has a chemosuppression of 100% (Table 6).

**Table 6.** Chemoprophylactic Activity of Methanolic Crude Extract of Stem Bark of *Combretum molle* Against *Plasmodium berghei* Infection in Mice.^*^,^†^

Test Dose	Average % Parasitemia	% Suppression	Mean Survival Time ± SEM (Days)
NC	39.40 ± 1.21	0.0	6.6 ± 0.4
CM 100 mg/kg	26.4 ± 0.48^a3^	32.99	8.40 ± 0.5
CM 200 mg/kg	21.2 ± 1.24^a3^	46.2	11 ± 0.7
CM 400 mg/kg	17.8 ± 0.86^a3b1^	54.8	12.8 ± 0.86^a3b1^
CQ 10 mg/kg	0.0 ± 0.0^a3b3^	100	28 ± 0.00^a3b3^

Abbreviations: CM, *Combretum molle*; CQ, chloroquine; NC, negative control; SEM, standard error of mean.

* Values are expressed as percentage and mean ± SEM (n = 5).

^†^ a, as compared to NC; b, to CM 100 mg/kg; c, to CM 200 mg/kg; d, as compared to CM 400 mg/kg; ^1^ *P* < .05; ^2^ *P* < .01; ^3^ *P* < .001.

The standard drug and the higher doses of the extract had shown to have a more significant survival effect as compared with the negative control and treated group receiving the lower doses of the extract (*P* < 0.05). However, the lower and the middle doses of the extract did not reveal a significant survival effect as compared with the negative control (*P* > 0.05; Table 6).

### Body Weight and PCV Variation in Prophylactic Test

In the prophylactic model, the percent change in weight and PCV of the different groups of mice was shown to be affected in a dose dependent manner. The average percent change in weight of mice receiving the crude extract at doses of 200 mg/kg and 400 mg/kg was significantly different as compared with the negative control (*P* < 0.05). Similarly, the average percent change in PCV of mice receiving all doses of the extract was significantly different as compared with the negative control (*P* < 0.05; Table 7).

**Table 7.** Effect of Crude Methanolic Extract of the Stem Bark of *Combretum molle* on Body Weight and PCV of *Plasmodium berghei* Infected Mice in Prophylactic Test.^*^,^†^

Test Dose	BW D0 ± SEM	BW D4 ± SEM	BW % Change	PCV5 D0 ± SEM	PCV8 D4 ± SEM	PCV % Change
Distilled H_2_O 0.2 mL	30.1 ± 0.9	27.8 ± 0.8	−7.64	52 ± 1.1	42.4 ± 2.2	−18.5
CM 100 mg/kg	28.3 ± 0.9	26.98 ± 1	−4.66	54 ± 0.71	48.8 ± 0.66	−9.6^a1^
CM 200 mg/kg	30.7 ± 1.7	29.8 ± 1.7	−2.93^a1^	51.4 ± 0.5	48 ± 0.63	−6.6^a2^
CM 400 mg/kg	28.9 ± 2.4	28.5 ± 2.5	−1.38^a2^	54.6 ± 1.5	53 ± 1.67	−2.9^a3^
CQ 10 mg/kg	30.4 ± 1.9	30.3 ± 2.1	−0.33^a3b1^	53.4 ± 2.7	51.5 ± 2.38	−3.3^a3^

Abbreviations: BW, body weight; CM, *Combretum molle*; CQ, chloroquine; NC, negative control; PCV, packed cell volume; SEM, standard error of mean.

* Values are expressed as percentage and mean ± SEM (n = 5).

^†^ a, as compared to NC; b, to CM 100 mg/kg; and ^1^ *P* < .05; ^2^ *P* < .01; ^3^ *P* < .001.

## Discussion

The aim of this study was to evaluate the antimalarial activity of the crude extract of the stem bark of *Combretum molle* in animals where the acute toxicity study is mandatory before performing the other antimalarial protocols. According to the acute toxicity study, there were no deaths and/or signs of toxicities observed, which give clues on the safety of the traditional plant on its antimalarial folklore use.

According to the chemosuppressive test, the crude extract was found to be effective for reducing the parasitemia in a dose-dependent manner. Though the chemosuppressive effect of the extract was not comparable to the standard drug, the 3 doses of the extract had been shown to inhibit the parasitemia of the mice significantly as compared with the negative control. This chemosuppressive effect of the plant was supported by the greater survival period of the mice treated especially by the higher and the middle doses of the extract as compared with that of the negative control. This chemosuppressive effect of the present medicinal plants is in line with that of *Asparagus africanus*.^33^

Malaria is known to suppress appetite and causes loss of body weight.^34^ In addition, the decline in the plasmodium parasite in the RBCs is associated with an increase in PCV value.^35^ In the present study, the percentage reduction in body weight and that of the PCV was very less in those mice taking the different doses of crude extract as compared with those mice receiving the vehicle only, which might be due to the antimalarial activity of the traditional plant on the early phases of malarial infection. The chemosuppressive test of the crude extract might indicate that the crude extract had affected the early infective stages of *Plasmodium berghei* in the prognosis of malarial diseases as a reduction in the parasitemia level is mandatory for preventing the development of symptomatic malaria.^30^

In the curative or Rane test, the administration of the crude extract at the tolerable doses reduced the average percentage of parasitemia progressively from the day after treatment to the end of treatment. The higher the dose of the extract given, the higher was its chemosuppressive effect across the duration of treatment, which revealed its dose-dependent curative effect in the pathogenesis of malaria. The dose-dependent antimalarial activity of the crude extract was assured by differences in the level of parasitemia of mice receiving different doses of the extract as it was tested at different time intervals as compared with those groups of mice taking the vehicle only. Similar to other studies,^32,36^ the plasmodial parasite inhibition effect in mice was strongly associated with the prolongation in the survival time of mice treated with the crude extract. The curative potential of the crude extract was supported by its significant prolonging of the survival of mice in a dose-dependent manner as compared with that of the negative control during the 30-day follow-up period. In addition, all doses of the extract had not caused a significant reduction in the PCV of treated mice as compared with that of the negative control. These findings could also strengthen the antiplasmodial activity of the crude extract of stem bark of *Combretum molle*.

The chemoprophylactic potential of the crude extract of the plant was also found to be effective at the given doses of the study. In the prophylactic test, the 3 doses of the extract was found to have a significant inhibition effect on the level of parasitemia of all treated groups as compared with the negative control. This prophylactic potential of the plant was supported by the prolonged survival of mice treated with the 3 doses of the crude extract as compared with the mice taking the vehicle only. In addition, the malaria preventive potential of the crude extract was also supported by the negligible reduction in body weight and PCV of treated mice as compared with the negative control in which percent change in body weight and PCV was significant.

Based on the qualitative screening test, the crude extract of *Combretum molle* was found to be positive for the presence of alkaloids, anthraquinone, terpenoids, flavonoids, and phenols, which have been considered to have antiplasmodial activity.^32,36–40^ The antimalarial activity of the plant might result from the action of these secondary metabolites that act individually or synergistically against the infection caused by plasmodium species.

The antimalarial activity of *Combretum molle* might be attributed to the presence of phenols, which are approved to have antioxidant activity against the damage or polymerization of red blood cells.^41^ The content of flavonoids, terpenoids, and alkaloids in the crude extract of the study plant might contribute to the chemosuppressive activity of the extract as they are suggested to have antiplasmodial activity.^30^ The antimalarial activity of flavonoids is suggested to be modulating the immune system of animals^42^ and having antioxidant activity.^43^ Alkaloids might contribute to the antimalarial activity of the study plant as quinine, one of the antimalarial drugs, belongs to alkaloids, which are also proven to have antioxidant activity.

The chemosuppressive, curative, and prophylactic antiplasmodial evaluations of the crude extract of the stem bark of *Combretum molle* could be of one the scientific evidence for the traditional antimalarial use of the plant in the community. Therefore, the health care policy of Ethiopia has to strengthen the integrated treatment approach of traditional medicine practice and modern medicine in order to effectively manage different diseases like malaria and to make use of the traditional medicinal plants as a potential source of new drugs for the scientific world.

## Limitations

Further antimalarial activity of the solvent fractions and identification of the antimalarial constituent(s) of the plant was not conducted as a result of budget and time constraints and due to the absence of well-equipped laboratory facilities. This could limit the expected contributions of this study to the antimalarial drug discovery and development process of the scientific communities. Therefore, the crude extract of *Combretum molle* has to be further studied by the fractional extraction method using different solvents that do vary in polarity. Then, each fraction can be characterized to identify the responsible compound(s) having antiplasmodial activity using sophisticated instruments like high-performance liquid chromatography hyphenated with spectrometric techniques. Then, the compound can be formulated in appropriate dosage forms and used as an experimental drug in preclinical and clinical trials, which may, in turn, lead to the development and discovery of new clinically effective antimalarial drugs.

## Conclusions and Recommendations

The findings of the present study has supported the folklore use of the stem bark of *Combretum molle* for the management of malaria. The antimalarial activity of the plant might be related to the presence of alkaloids, flavonoids, phenols, terpenoids, and flavonoids, which might act in single or in combination against *Plasmodium berghei*. The antiplasmodial findings of this study can be used as a baseline and give a clue to the scientific researchers who intend to discover and develop potential new antimalarial drugs. Therefore, further fractionation, isolation, and characterization of the crude extract is necessary to identify the responsible lead compound(s) with the potential of antiplasmodial activity.
